# An age–period–cohort–interaction analysis of meth/amphetamine‐related deaths in Australia, 2001–2020

**DOI:** 10.1111/add.70100

**Published:** 2025-07-01

**Authors:** Oisin Stronach, Paul Dietze, Michael Livingston, Amanda Roxburgh

**Affiliations:** ^1^ School of Public Health and Preventive Medicine, Faculty of Medicine, Nursing and Health Sciences Monash University Melbourne Australia; ^2^ Harm and Risk Reduction Program Burnet Institute Melbourne Australia; ^3^ National Drug Research Institute Curtin University Perth Australia

**Keywords:** age period cohort analysis, amphetamine, epidemiology, methamphetamine, mortality, overdose, toxicity

## Abstract

**Background and Aims:**

The number of meth/amphetamine related deaths in Australia has quadrupled in the last 20 years, primarily due to drug toxicity and suicide among individuals in their 30s and 40s. Previous analysis of Australian meth/amphetamine‐related deaths covered limited timeframes and causes, and there has been no exploration of the effects of changing cohorts on meth/amphetamine mortality. This paper provides comprehensive insights across 20 years into the evolving cohort trends in meth/amphetamine‐related deaths in Australia.

**Methods:**

An age–period–cohort–interaction (APC‐I) analysis of Australian meth/amphetamine‐related deaths (2001–2020) by cause extracted from the National Coronial Information System, a database of all deaths reported to the coroner in Australia and New Zealand.

**Results:**

APC‐I analyses revealed that unintentional drug toxicity deaths peaked at ages 35–38 [Coefficient (Coef) = 0.92; 95% confidence interval (CI) = 1.0–0.8], intentional self‐harm deaths peaked at ages 31–34 (Coef = 1.2; 95% CI = 1.4–1.0), unintentional injury deaths peaked at ages 23–26 (Coef = 1.02; 95% CI = 1.2–0.8) and natural cause deaths at 39–42 (Coef = 1.15; 95% CI = 1.4–0.9). Period effects were consistent across all causes, with a mean 29.3% increase in estimated mortality rate from 2001 to 2012, followed by a mean 103.3% increase in estimated mortality rate to 2016, at which time period effects stabilised. Cohort effects revealed that individuals born between 1962 and 1982 (mainly Generation X) faced a higher‐than‐average mortality risk across all four causes, with risk decreasing in later generations.

**Conclusions:**

Despite different age profiles across the various causes of death, cohort effects suggest a single generation (Generation X: people born between 1962 and 1982) is predominantly experiencing the increase in meth/amphetamine‐related mortality observed in Australia over the past 20 years. As Generation X ages, the risk of meth/amphetamine‐related natural deaths, especially from cardiovascular disease, is likely to increase.

## INTRODUCTION

Australia is estimated to have one of the highest rates of people experiencing amphetamine dependence in the world [1, 2]. The 2022 to 2023 Australian National Drug Strategy Household Survey (NDSHS) estimates that 7.5% of Australians have used methamphetamine and/or amphetamine in their lifetime, (hereafter meth/amphetamine), whereas an estimated 1% used it in the past year and 0.3% used it weekly [3]. Although reports of past‐year use of meth/amphetamines have decreased over time, frequency of use among people reporting past‐year use has increased [4]. Among people aged 40 years and older, meth/amphetamine use appears to be increasing, while it is simultaneously decreasing among younger people, suggesting there are potential cohort patterns [4, 5]. It is unclear if these trends stem from people who regularly use meth/amphetamine ageing, or if younger individuals are becoming more aware of meth/amphetamine‐related harms [6]. Over the past two decades, meth/amphetamine‐related mortality in Australia has quadrupled, primarily from drug toxicity and suicide among individuals in their 30s and 40s [7], with meth/amphetamine‐related drug toxicity deaths peaking in 2020 (2.5 deaths per 100 000 population) [8].

When analysing trends in meth/amphetamine‐related deaths, there are three associated, time‐related effects that influence longitudinal changes: age, period and cohort (APC) effects. Age effects refer to changes that occur as an individual ages and are attributed to the ageing process itself, regardless of birth year [9]. Period effects impact all age groups simultaneously because of external factors/events occurring during a specific period. They can arise from societal, economic, environmental or technological changes affecting everyone, regardless of age [9]. Cohort effects are enduring impacts of historical events/social norms at specific times that affect people differently based on age [9].

The sociological conceptualisation of cohorts suggests all people born within the same period and physical location are exposed to a specific range of experiences, predisposing them to thinking and behaving in certain ways [10]. Experiences are particularly influential during key developmental stages, such as adolescence and young adulthood [11]. Ryder [12] suggested cohort effects can change over time because they are constantly being shaped by significant life events and social structures and can significantly influence a cohort's health and mortality patterns. Previous APC methods assumed these effects were constant across ages [13, 14, 15, 16, 17, 18]; however, in line with Ryder's [12] notion of cohorts, recent APC research has suggested that cohort effects may change across a person's lifespan [19, 20].

In the United States (US), APC research has found there has been a generational shift in drug overdose (including meth/amphetamine) mortality to younger, non‐white populations with each ‘wave’ of the overdose crisis [21]. APC research also indicates the historical trend of declining rates of high‐risk meth/amphetamine use with age no longer holds for individuals born after 1990 [22]. Unlike North America, fentanyl‐related deaths in Australia are relatively infrequent and largely attributable to pharmaceutical fentanyl [23]. Consistent with all drug‐related mortality in Australia, fentanyl‐related mortality predominantly occurs among older Australians, with declining rates reported among younger Australians [8, 23]. This trend is consistent with other trends in drug use and harms, such as the observed increasing age of initiation of injecting drug use recorded in Australia [24].

APC analysis has not been applied to meth/amphetamine‐related deaths in Australia, and no APC analysis of meth/amphetamine‐related deaths stratified by cause of death exists internationally. Findings from APC analyses may help public health efforts to reduce and prevent drug‐related deaths. In light of shifts in drug markets, evolving consumer practices and changing age‐specific mortality patterns, this study extends previous longitudinal trend research [7, 8, 25, 26] to provide an Australian analysis of APC effects over a 20‐year period from 2001 to 2020 by cause of death.

### Aims

This study aims to (1) describe the APC patterns of Australian meth/amphetamine‐related mortality between 2001 and 2020; and (2) determine which cohorts have a high or low risk of meth/amphetamine‐related mortality relative to their age or period.

## METHODS

### Design

This study uses the age–period–cohort–interaction (APC‐I) model developed by Luo and Hodges [19], and follows the methodology for its application with aggregate‐level data outlined by Lu and Luo [27] to gain insights into variations in APC effects among meth/amphetamine‐related deaths by cause. This approach estimates age and period effects as well as cohort effects that are the product of the age‐by‐period interaction, which allows the cohort effects to vary by age [19].

### Data

Data was obtained from the National Coronial Information System (NCIS), an on‐line national database containing information on all ‘reportable deaths’ in Australia from 1 July 2000 (Queensland from 1 January 2001) and New Zealand from 1 July 2007. A death must be reported to the coroner if it is unforeseen, unexplained, caused by an accident or injury, occurs while the individual is in care or custody or involves an unknown identity [28]. In this study, only deaths involving illicit meth/amphetamine were included, identified using keyword searches of the medical cause of death fields and mechanism of injury coding. This includes deaths involving meth/amphetamine in conjunction with other drugs. Cases were then categorised by cause of death as determined by the coroner. The manner of death was classified as (1) unintentional drug toxicity; (2) intentional self‐harm (including toxicity); (3) natural causes; (4) unintentional injury; and (5) assault (see Table S1 for definitions). Assault deaths were excluded because of low numbers.

Deaths were grouped into five 4‐year periods between 2001 and 2020, with each period denoted by the latter middle year (e.g. 2001–2004 was coded as 2003). Four‐year periods were chosen to balance the need for temporal resolution while retaining sufficient data in each period. Age at death was categorised into 4‐year groups, denoted by the latter middle year. This category includes deaths from age 17 (15‐ to 18‐year‐olds) to the top category of 61 (59‐ to 62‐year‐olds). Cohorts were defined as period minus age, for example deaths for individuals aged 15 to 18 [17] in the period 2001 to 2004 (2003) were assigned the cohort 1986. Cohorts were then grouped by generation names (‘Baby Boomers’ born between 1946 and 1964; ‘Generation X’, 1965–1980; ‘Millennials’, 1981–1995; and ‘Generation Z’, 1996–2015). Population estimates for each period were summed to estimate crude mortality rates. Crude mortality rates (per 100 000 population) were calculated for each combination of age and period, using the latest Australian Bureau of Statistics population estimates from 30 June of each year [29]. Although Australian generational cohorts are labelled similarly to those internationally, the defining experiences of these groups are influenced by Australia's specific historical events, cultural shifts, societal changes and importantly for this paper, trends in local drug markets. Each birth cohort grows up in a unique historical context, leading to distinctive trajectories shaped by the social norms and institutions of their period [30].

### Statistical analysis

#### Data visualisation

We used Lexis diagram hexamap visualisations before undertaking the APC analysis. The use of hexamaps for APC data visualisation overcomes the visual distortions in a traditional Lexis diagram [31]. The hexamaps are organised into hexagonal tiles, with age categories on the vertical axis and periods on the horizontal axis. Each grid cell is colour‐coded to represent crude mortality rates per 100 000 population for each cause of death, from light shades for low rates to dark shades for high rates. Cohorts are represented along diagonal lines, and a diagonal intensification of colour is indicative of possible cohort effects.

#### APC models

Standard APC models encounter limitations because of the identification problem, which stems from the linear relationship between the three variables: age, period and cohort (i.e. cohort = period ‐ age), leading to an exact collinearity among these factors. Consequently, when linear models try to incorporate all three variables simultaneously, they encounter a fundamental constraint, as they produce an infinite array of potential solutions for the coefficients [32]. This collinearity issue complicates the model's capacity to delineate and separate the individual contributions of APC effects, limiting the explanatory power of such models. Researchers have posed a range of methods to resolve the identification problem [13, 14, 15, 16, 17, 18], but as summarised by Bell [33], these methods all rely on implicit or explicit assumptions that challenge interpretation. One key assumption underlying most APC methods is that there are independent and additive effects of APC. This assumes cohort effects operate independently of age and social change, and ignores that cohort effects may vary over a person's life, which does not align with the theoretical conceptualisation of cohort effects [12].

#### APC‐I models

The primary analysis focused on examining meth/amphetamine mortality using the APC‐I model [19], which is a method that addresses the inherent limitations of traditional APC analysis. Full details of the statistical modelling approaches used here are published elsewhere [19, 27, 34], but briefly, the APC‐I approach differs from the APC models listed above because the age and period effects are modelled as the main effects, and cohort effects are products of the age‐by‐period interaction term. The APC‐I model in its general form can be specified as a generalised linear model as follows:

$$g\left(E\left(Y_{\mathrm{ij}}\right)\right)=\mu +\alpha_{i}+\beta_{j}+\alpha \beta_{\mathrm{ij}\left(k\right)}$$

$\mathrm{gE}\left(Y_{\mathrm{ij}}\right)$ is the link function of the expected mortality rate Y for the i‐th age group in the j‐th period, μ denotes the global mean of the observed mortality rates, $\alpha_{i}$ represents the main age effects associated with the i‐th age category, $\beta_{j}$ represents the main period effects associated with the j‐th period, and $\alpha \beta_{\mathrm{ij}\left(k\right)}$ denotes the interaction of the i‐th age group and j‐th period group, corresponding to the effect of the k‐th cohort. Note that the cohort effect includes multiple $\alpha \beta_{\mathrm{ij}\left(k\right)}$ interaction terms for each observation on a diagonal in a table with ages in rows and periods in columns [19].

This method allows for cohort‐specific dynamics reflecting unique life‐course impacts as a person ages, rather than assuming static, additive cohort effects. The APC‐I model is methodologically innovative as it provides a statistical framework to analyse the difference between cohorts (inter‐cohort differences) and variations within the same cohorts (intra‐cohort deviations). It evaluates how cohort effects deviate from average age and period effects, and assesses within‐cohort variations against expected patterns based on age and period.

We use a sum‐to‐zero encoding strategy for all variables, known as effect coding. This methodology enables the model coefficients to represent deviations from the total mean rather than deviations from an excluded category, which facilitates the interpretation of the APC‐I model. First, a global deviance test was undertaken for each cause of death, where a generalised linear model (GLM) with age and period terms (an AP model) was tested against a GLM that included age, period and age‐by‐period terms (an APC‐I model), using a likelihood ratio test. The AP and APC‐I models were fitted with Poisson regression (a full justification for using Poisson AP and APC‐I models can be found in the Supporting information and Figure S1). A significant result suggested that the APC‐I model provided a better fit for the data and cohort effects may be present. Second, we fitted separate GLM APC‐I models for each cause of death that included the age and period main effects. Third, we investigated inter‐cohort differences by calculating the average of the age‐by‐period interaction terms for each cohort and then conducted a z test to determine if it was significantly different from the expected rate based on the age and period main effect. A positive value suggested a cohort has a higher mortality rate than the predicted rate determined by only the age and period effects. A significant cohort effect represents higher‐ or lower‐than‐expected mortality relative to the overall age and period effects. The APC‐I approach uniquely allows cohort effects to vary with age, distinguishing it conceptually and methodologically from other APC models where cohort effects are traditionally assumed to be static. This step excludes the oldest (1942) and youngest cohort (2002), because only one age‐by‐period interaction for these cohorts can be observed. The final step involved assessing intra‐cohort trends by modelling the age‐by‐period interaction terms as a function of age using linear orthogonal polynomial contrasts and testing the significance of the intra‐cohort slopes via a z test. Given our analysis uses population data of all Australian meth/amphetamine‐related deaths, and statistical inferences are not being made outside of the study period, statistical significance is included solely for reference purposes. The focus of the study is effect sizes, and interpreting them as population parameters rather than sample estimates. The analyses were performed using the R statistical software (version 4.0.2), using custom scripts for the application of the APC‐I model for population data as outlined by Lu and Luo [27]. The analysis was not pre‐registered, and the results should be considered exploratory.

### Ethics

Ethics approval for this study was received from the Alfred Ethics Committee (61044) and from the Justice Human Research Ethics Committee (CF/20/4121) for the use of NCIS data.

## RESULTS

### Age and period effects

**FIGURE 1:**
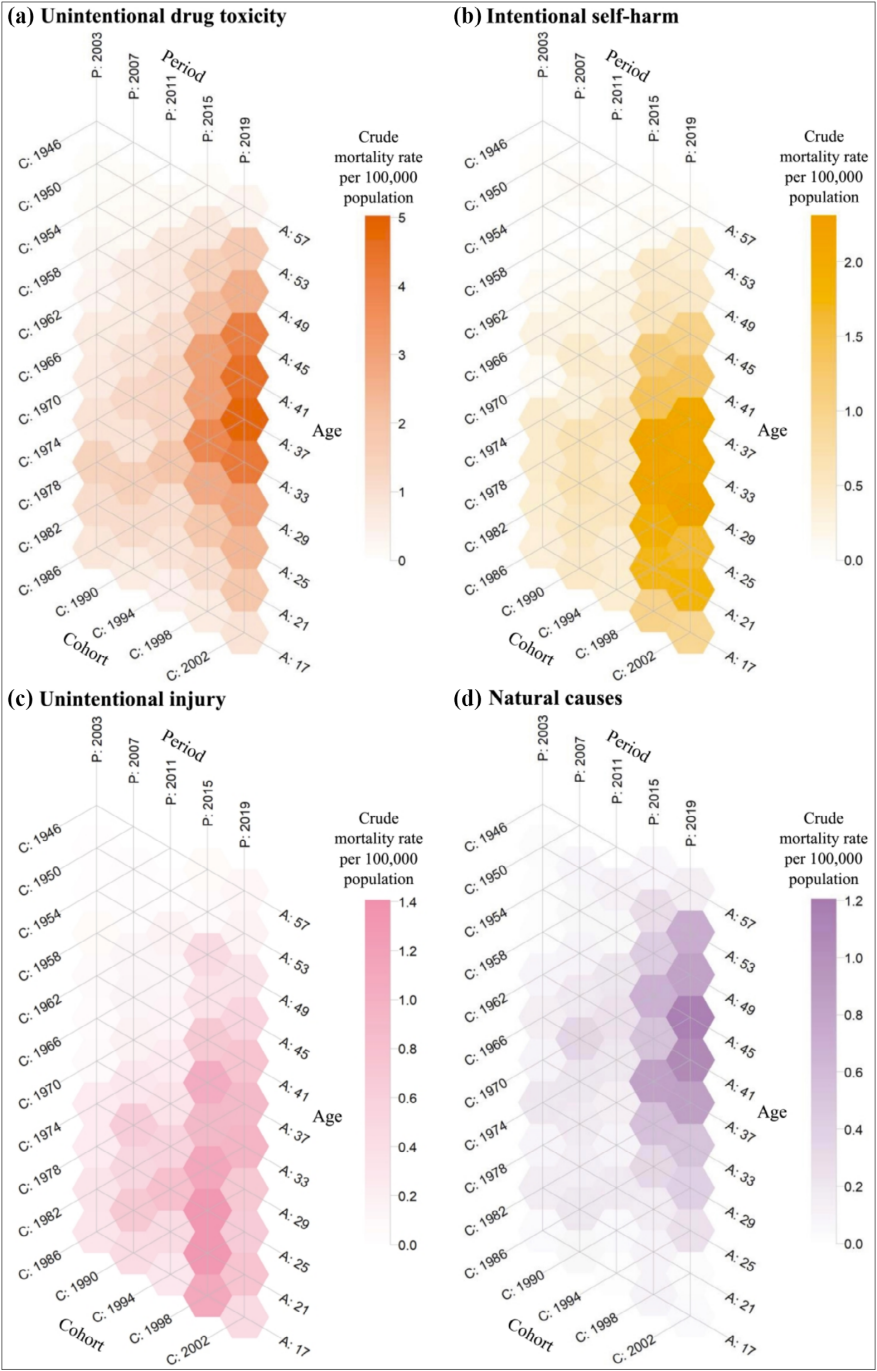
Lexis diagram hexamap of meth/amphetamine‐related crude mortality rates per 100 000 population by age, period and cohort for (a) unintentional drug toxicity, (b) intentional self‐harm, (c) unintentional injury and (d) natural causes.

Figure 1 shows rates of meth/amphetamine‐related mortality broken down by age group across study periods by cause of death, indicating a diagonal intensification of mortality rates across the study period for each cause. The hexamaps show a wedge shape that is largely consistent across all causes of death, starting with the 19‐ to 22‐year‐olds and finishing with the 1958 cohort (the diagonal cohort starting among 43‐ to 46‐year‐olds in 2001–2004). Figure S2 shows the peak age at death has increased over time for unintentional drug toxicity, intentional self‐harm and natural causes, whereas the peak age of unintentional injury has remained among younger age groups, with slight fluctuations.

Based on the hexamaps indicating potential cohort effects for each of the causes of death, we fitted two models that included (1) only age and period main effects and (2) all APC‐I interaction terms for all four causes. Table S2 shows that the more complex APC‐I model better fit the data and that cohort effects may be present for all four causes of death.

Table 1 displays age and period main effect estimates by causes of death. We found significant associations for most ages and periods. The intercept term serves as an estimator for the global mean mortality rate for each cause of death. The estimated age effect for 15‐ to 18‐year‐olds was −1.59, which suggests that this age group experiences 80% [1‐exp (−1.59)] lower in rates of meth/amphetamine‐related unintentional drug toxicity compared to the global mean. The estimated age effect for 35‐ to 38‐year‐olds was 0.92, which suggests that this age group experiences 151% [exp (0.92)‐1] higher rates of meth/amphetamine‐related unintentional drug toxicity compared to the global mean. It is important to note that significant associations may be influenced by low values in certain age groups.

**TABLE 1:** Estimated age and period main effects on meth/amphetamine mortality using APC‐I models.

	Unintentional drug toxicity		Intentional self‐harm		Unintentional injury		Natural causes	
	Coef. (95% CI)	%	Coef. (95% CI)	%	Coef. (95% CI)	%	Coef. (95% CI)	%
Intercept	**−18.77**		**−19.70**		**−19.89**		**−20.55**	
Age main effects								
15–18	**−1.59 (−1.9 to −1.2)**	**−79.6**	**−0.90 (−1.4 to −0.4)**	**−59.3**	−0.17 (−0.5 to 0.1)	−15.6	**−1.69 (−2.5 to −0.9)**	**−81.5**
19–22	−0.03 (−0.2 to 0.1)	−3.0	**0.74 (0.5–0.9)**	**77.1**	**0.71 (0.5–0.9)**	**74.8**	**−0.94 (−1.5 to −0.3)**	**−60.9**
23–26	**0.52 (0.4 to 0.6)**	**61.9**	**1.01 (0.8–1.2)**	**101.0**	**1.02 (0.8–1.2)**	**102.0**	−0.24 (−0.7 to 0.2)	−21.3
27–30	**0.82 (0.70.9)**	**83.5**	**1.15 (1.0–1.3)**	**116.2**	**0.99 (0.8 to 1.2)**	**99.0**	0.29 (0.0–0.6)	49.2
31–34	**0.88 (0.8–1.0)**	**88.7**	**1.20 (1.0–1.4)**	**122.1**	**0.96 (0.8–1.1)**	**96.1**	**0.63 (0.3 to 0.9)**	**69.1**
35–38	**0.92 (0.8–1.0)**	**92.3**	**0.84 (0.6–1.1)**	**85.2**	**0.55 (0.3–0.8)**	**63.8**	**0.74 (0.4–1.0)**	**77.1**
39–42	**0.83 (0.7–1.0)**	**84.4**	**0.88 (0.7–1.1)**	**88.7**	**0.35 (0.1–0.6)**	**52.2**	**1.15 (0.9–1.4)**	**116.2**
43–46	**0.57 (0.4–0.7)**	**65.1**	**0.39 (0.1–0.6)**	**54.3**	0.15 (−0.1 to 0.4)	42.7	**0.90 (0.6–1.2)**	**90.5**
47–50	**0.37 (0.20.5)**	**53.3**	−0.12 (−0.5 to 0.2)	−11.3	−0.18 (−0.5 to 0.1)	−16.5	**0.58 (0.1–1.0)**	**65.7**
51–54	−0.14 (−0.4 to 0.1)	−13.1	**−1.05 (−1.6 to −0.5)**	**−65.0**	**−0.60 (−1.1 to −0.1)**	**−45.1**	0.10 (−0.4 to 0.6)	40.7
55–58	**−0.88 (−1.2 to −0.5)**	**−58.5**	**−1.31 (−1.9 to −0.7)**	**−73.0**	**−1.38 (−2.0 to −0.8)**	**−74.8**	0.14 (−0.4 to 0.6)	42.3
59–62	**−2.27 (−2.6 to −2.0)**	**−89.7**	**−2.84 (−3.4 to −2.2)**	**−94.2**	**−2.4 (−3.0 to −1.8)**	**−90.9**	**−1.67 (−2.3 to −1.1)**	**−81.2**
Period main effects								
2001–2004	**−0.74 (−0.9 to −0.6)**	**−52.3**	**−1.07 (−1.4 to −0.8)**	**−65.7**	**−0.92 (−1.2 to −0.6)**	**−60.1**	**−0.81 (−1.2 to −0.5)**	**−55.5**
2005–2008	**−0.45 (−0.6 to −0.3)**	**−36.2**	**−0.38 (−0.6 to −0.2)**	**−31.6**	**−0.36 (−0.6 to −0.1)**	**−30.2**	−0.24 (−0.5 to0.0)	−21.3
2009–2012	**−0.26 (−0.4 to −0.1)**	**−22.9**	**−0.50 (−0.8 to −0.3)**	**−39.3**	**−0.24 (−0.5 to 0.0)**	**−21.3**	**−0.40 (−0.7 to −0.1)**	**−33.0**
2013–2016	**0.50 (0.4–0.6)**	**60.7**	**0.86 (0.7–1.0)**	**86.9**	**0.84 (0.7–1.0)**	**85.2**	**0.66 (0.4 to 0.9)**	**71.2**
2017–2020	**0.95 (0.9–1.0)**	**95.1**	**1.08 (0.9–1.2)**	**108.3**	**0.68 (0.5–0.8)**	**72.6**	**0.80 (0.6–1.0)**	**81.9**

*Note*: Bold indicates significant deviations from the global mean. Given that our analysis uses population data for all Australian methamphetamine‐related deaths and statistical inferences are not being made outside of the study period, statistical significance is included solely for reference purposes. We focus on the effect sizes, interpreting them as population parameters rather than sample estimates.

Abbreviations: APC‐I, age‐period‐cohort‐interaction; Coef., coefficient.

**FIGURE 2:**
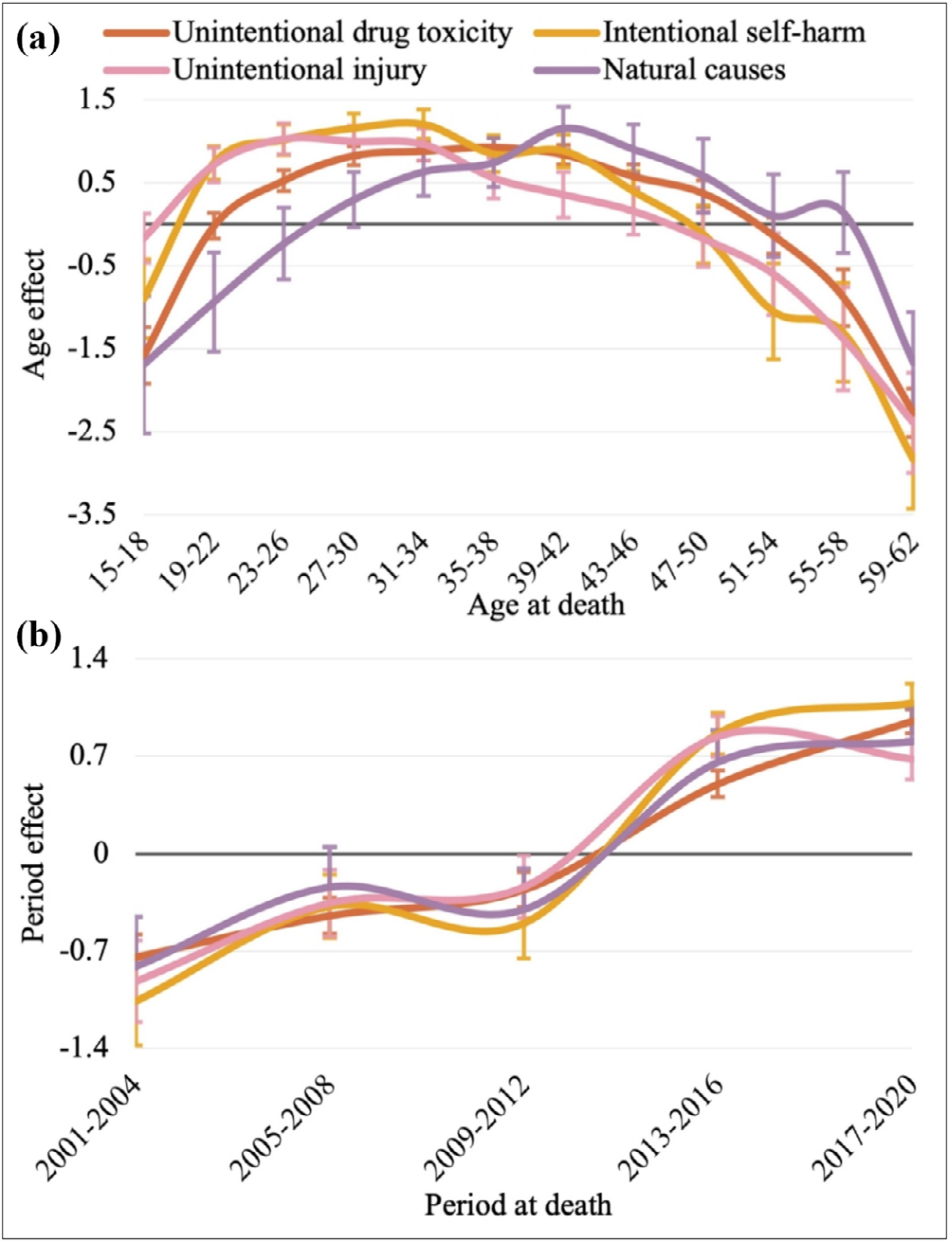
Estimated age (a) and period (b) main effects of meth/amphetamine‐related unintentional drug toxicity, intentional self‐harm, unintentional injury and natural causes deaths. The horizontal solid line represents zero deviation from the global mean [intercept of the age‐period‐cohort‐interaction (APC‐I) model]. Data points positioned above this baseline indicate higher‐than‐average risks of mortality and points located below the baseline denote lower‐than‐average risks of mortality.

Figure 2(a) graphically represents the age main effect shown in Table 1, after adjusting for period effects. People aged between 23 and 50 years old had significantly higher unintentional drug toxicity rates compared to the global mean, with a peak of 92.3% increased mortality risk between ages 35 to 38 [coefficient (Coef) = 0.92; 95% CI = 0.8–1.0]. People aged 19 to 46 had higher rates of intentional self‐harm, with a peak of 122.1% increased mortality risk between ages 31 to –34 (Coef = 1.2; 95% CI = 1.0–1.4). People between 19 and 42 years old had higher rates of unintentional injury deaths, with a peak of 102.0% increased mortality risk between ages 23 to 26 (Coef = 1.02; 95% CI = 0.8–1.2). For natural causes, individuals aged 31 to 50 had higher mortality, with a peak of 116.2% increased mortality risk between ages 39 to 42 (Coef = 1.15; 95% CI = 0.9–1.4).

We interpret the period main effects in Table 1 as percentage deviations from the global mean, the same way as the age main effects. Figure 2(b) graphically represents the period main effect shown in Table 1. After adjusting for age effects, period effects for all causes of death follow a similar pattern with significantly higher mortality rates between 2013 and 2020. Unintentional injury deaths peak between 2013 to 2016, with an 85.2% (Coef = 0.84; 95% CI = 0.7–1.0) increased mortality risk. Unintentional drug toxicity, intentional self‐harm and natural cause deaths period effects peak in 2017 to 2020, with a 95.1% (Coef = 0.95; 95% CI = 0.9–1.0), 108.3% (Coef = 1.08; 95% CI = 0.9–1.2) and 81.9% (Coef = 0.8; 95% CI = 0.6–1.0) increased mortality risk, respectively.

### Cohort effects

Table 2 represents inter‐cohort deviations and intra‐cohort slopes for meth/amphetamine‐related mortality rates by cause of death. Inter‐cohort deviations are based on mean age‐by‐period interaction terms (see Tables S3.1–S3.4) of each cohort across all periods. Inter‐cohort deviations indicate how each birth cohort's observed mortality rate across the study period differs, on average, from what would be expected based on predicted age and period terms. For example, the 1978 cohort had the highest inter‐cohort deviation among unintentional drug toxicity deaths of 0.37. This means that the average meth/amphetamine‐related unintentional drug toxicity mortality rate for that cohort (Generation X) was 45% higher than the average cohort. Intra‐cohort slopes show how the cohort's mortality risk changes as they age, with positive slopes suggesting increasing relative risk over time and negative slopes indicating decreasing relative risk as the cohort ages.

**TABLE 2:** Estimated inter‐cohort deviations and intra‐cohort life‐course dynamics for meth/amphetamine‐related unintentional drug toxicity, intentional self‐harm, unintentional injury and natural causes mortality.

Cohort	Unintentional drug toxicity		Intentional self‐harm		Unintentional injury		Natural causes		Generation
	Inter‐cohort Coef.	Intra‐cohort slope	Inter‐cohort Coef.	Intra‐cohort slope	Inter‐cohort Coef.	Intra‐cohort slope	Inter‐cohort Coef.	Intra‐cohort slope	
1942	−0.20	N/A	0.23	N/A	−0.16	N/A	−0.34	N/A	
1946	−0.56	0.51	0.30	0.33	−0.33	−0.73	−0.91	−0.17	Baby Boomers
1950	**−0.44**	0.33	−0.17	−0.18	−0.30	0.24	−0.52	0.41	
1954	**−0.28**	**0.55**	−0.61	0.42	−0.26	0.30	−0.22	**1.53**	
1958	−0.09	0.20	−0.10	0.09	−0.14	−0.72	**−0.42**	−0.22	
1962	**0.16**	**0.84**	**0.34**	**0.56**	0.03	**0.82**	**0.29**	**0.89**	
1966	0.10	**0.60**	0.00	**0.71**	−0.13	0.46	**0.39**	**0.79**	Generation X
1970	**0.22**	**0.29**	0.11	0.18	0.13	0.22	**0.31**	0.03	
1974	**0.18**	−0.06	**0.15**	−0.23	**0.25**	0.20	0.14	0.19	
1978	**0.37**	**−0.36**	**0.16**	−0.12	**0.19**	−0.03	**0.28**	**−0.89**	
1982	**0.22**	**−0.75**	**0.16**	−0.36	**0.30**	**−0.44**	**0.44**	**−0.95**	Millennials
1986	**0.16**	**−1.17**	−0.14	0.10	0.07	**−0.93**	0.09	−1.08	
1990	−0.14	**−0.90**	0.02	**−0.71**	−0.16	**−0.56**	−0.27	−0.43	
1994	**−0.53**	0.04	−0.11	−0.36	−0.08	**−0.54**	−0.45	−1.56	
1998	**−0.54**	−0.12	−0.41	−0.28	**−0.58**	−0.16	−0.84	−0.23	Generation Z
2002	**−0.78**	N/A	−0.04	N/A	**−0.80**	N/A	−0.83	N/A	

*Note*: Bold indicates significant deviations.

Abbreviation: Coef., coefficient.

**FIGURE 3:**
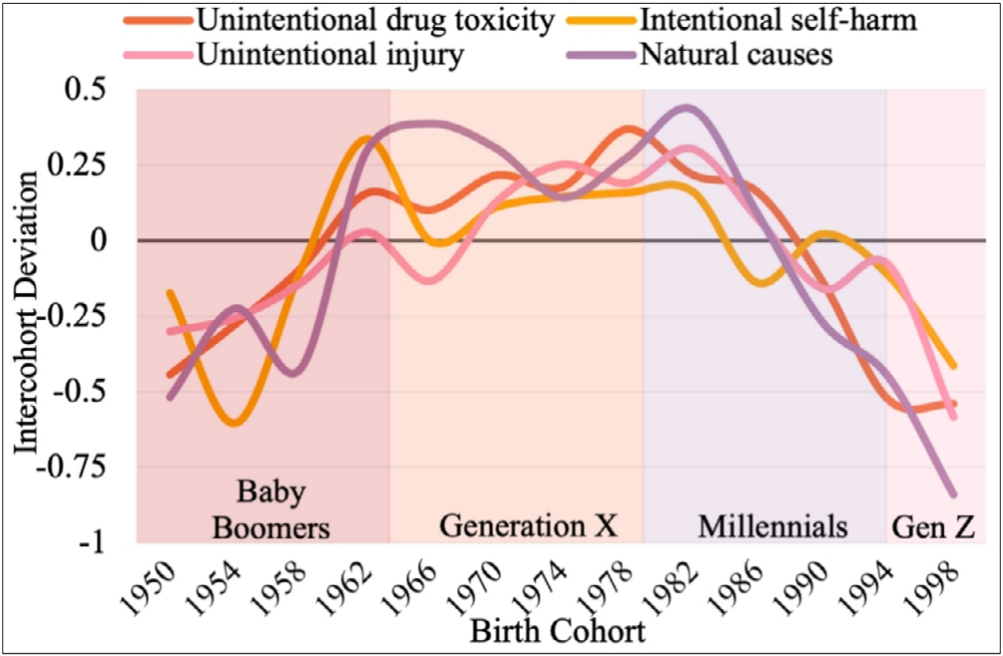
Estimated inter‐cohort deviations of meth/amphetamine‐related unintentional drug toxicity, intentional self‐harm, unintentional injury and natural causes deaths. The horizontal solid line represents no deviation from the predicted mortality rate determined by age and period main effects. Data points positioned above this baseline indicate cohorts with higher‐than‐average risks of mortality and points located below the baseline indicate cohorts with lower‐than‐average risks of mortality.

Overall trends in inter‐cohort deviations can be seen in Figure 3. Although there were slight variations by cause of death, inter‐cohort deviations largely follow the same pattern. Among unintentional drug toxicity‐related deaths, negative inter‐cohort effects were observed among the early and mid‐Baby Boomer cohorts of 1946 (−0.56), 1950 (−0.44) and 1954 (−0.28). Positive effects were observed among late‐Baby Boomer, Generation X and early Millennial cohorts of 1962 (0.16), 1966 (0.10), 1970 (0.22), 1974 (0.18), 1978 (0.37), 1982 (0.22) and 1986 (0.16). Inter‐cohort effects became negative among the late‐Millennial and Generation Z cohorts of 1990 (−0.14), 1994 (−0.53), 1998 (−0.54) and 2002 (−0.78). Inter‐cohort trends were similar among intentional self‐harm, unintentional injury and natural causes.

Table 2 shows that mortality risk for each cohort varied with age and period. The unintentional drug toxicity birth cohorts of 1962 and 1970 had positive inter‐cohort deviations (0.16 and 0.22, respectively) and a significant positive intra‐cohort slope (0.84 and 0.29, respectively), suggesting a higher than expected mortality risk, relative to their age, with risk increasing as they got older. In contrast, the unintentional drug toxicity cohorts of 1978, 1982 and 1986 had positive inter‐cohort deviations (0.37, 0.22 and 0.16, respectively) and a significant negative intra‐cohort slope (−0.36, −0.75 and −1.17, respectively), suggesting a higher than expected mortality risk, relative to their age, with risk decreasing as they got older. These trends are graphically shown in Figure S3 and are similar across the causes of death.

## DISCUSSION

We applied an APC‐I model to meth/amphetamine‐related deaths in Australia. The shifting age‐specific mortality patterns, and the move toward older age groups, suggest the influence of broader cohort factors. Similar trends are evident in meth/amphetamine use, with the median age of use increasing from 23.1 in 2001 to 32.1 in 2019 [4]. These trends are in contrast with age patterns of overdose in the United States where they have seen a shift toward younger age groups [21].

### Age effects

There were variations in age effects between the causes of death. Unintentional drug toxicity age effects showed mortality risk peaking among people aged 35 to 38 years old. Mortality risk for intentional self‐harm peaked among people aged 31 to 34, younger than that observed for opioid‐related intentional self‐harm deaths in Australia (peaking among 35‐ to 44‐year‐olds in 2019) [35]. Mortality risk for unintentional injury (largely driven by motor vehicle collisions) was higher among younger age groups (23‐ to 26‐year‐olds). This is consistent with data on all Australian motor vehicle collisions where the highest mortality rate in 2022 was among 17‐ to 25‐year‐olds (8.0 per 100 000 population in 2022) [36]. Unsurprisingly, mortality risk for natural causes was highest among older ages (39‐ to 42‐year‐olds). This pattern may be attributed to the prevalence of cardiovascular diseases as the leading natural cause in Australian meth/amphetamine‐related deaths [7], where risk escalates with age [37].

### Period effects

Period effects followed similar patterns across all causes of death. A negative period effect was evident between 2001 and 2012, followed by a positive effect between 2013 and 2020. Unintentional injury was the only cause associated with decreased deaths between 2017 and 2020. Increases in period effects align with the changing patterns of meth/amphetamine use, with an increase in regular use of high purity crystal meth/amphetamine across different populations [1, 2, 38]. The cost of purchasing meth/amphetamine increased in parallel with rising purity until 2011 to 2012, when both price and purity stabilised [38]. As the availability of high‐purity crystal meth/amphetamine increased, the cost decreased between 2015 and 2016 [6]. The increased availability of lower‐cost crystal meth/amphetamine may have contributed to a rise in dependence on meth/amphetamine and an increase in the adverse effects associated with its use, including mortality [39, 40, 41].

### Cohort effects

Our findings regarding cohort deviations should be interpreted relative to expected mortality derived from age and period main effects. Therefore, positive cohort effects indicate higher‐than‐expected mortality, highlighting unique risk dynamics associated with specific birth cohorts as they age. The inter‐cohort effects were largely consistent across all causes of death. Positive cohort effects were evident from late Baby Boomers to early Millennials (cohorts 1962–1982), but were mainly among Generation X. Generation X was aged between 20 and 35 during the Australian heroin ‘shortage’ in late 2000/early 2001, where the availability and purity of heroin decreased dramatically. Disruption to supply continued to have an impact on the purity of heroin for several years [42, 43, 44]. During this period injecting initiation with meth/amphetamine increased and is associated with an increased prevalence of injecting a combination of heroin and meth/amphetamine later in life [45]. Meth/amphetamine powder (speed) use was highly prevalent among people who injected drugs and was the drug commonly consumed when initiating injecting drug use at this time [46, 47]. The change in the drug market and prominent use of meth/amphetamine powder in the early 2000's followed by the market transition to crystal meth/amphetamine in the early 2010's may have had a unique effect on Generation X's mortality risk [48].

Despite late Baby Boomers, Generation X and early Millennials each having higher than average mortality risks, intra‐cohort effects suggest different trends among these generations. Late Baby Boomer cohorts experienced an increased risk of mortality, relative to their age, as they got older. Generation X experienced a high but stable risk of mortality relative to their age, as they got older. Early Millennials experienced decreased risk of mortality relative to their age as they got older. The decreased mortality risk among Millennials could indicate that treatment services have successfully engaged with this generation, as they made up the majority of clients who received treatment for meth/amphetamine use between 2018 and 2019 [48].

The lower meth/amphetamine mortality risk observed among younger generations is part of a broader trend of decreasing risk behaviours among young people seen in Australia and internationally [49]. This trend includes reductions in smoking, alcohol consumption, early sexual initiation and crime [49, 50, 51, 52]. Several factors are believed to influence these trends, including improved education about associated risks, stricter substance regulations [53, 54] and the impact of social media, which may spread positive health messages or provide a platform for peer influence [55, 56, 57]. Public health campaigns also contributed to young people viewing meth/amphetamine as a dangerous ‘problem drug’ [58], exacerbating stigma [59]. Between 2001 and 2019, the age of meth/amphetamine initiation increased from 20.4 to 22.5 years, and the percentage of 14‐ to 19‐year‐olds who reported past year meth/amphetamine use dropped from 6.2% in 2001 to 0.9% in 2019 [4]. Declines in meth/amphetamine use and mortality among young Australians is in contrast with the rising youth mortality in the United States, where younger generations are engaging in riskier drug use patterns in a more saturated drug market, including increased meth/amphetamine and opioid co‐use [22, 60].

### Policy implications

The increasing age of those using and experiencing harms related to meth/amphetamines may necessitate age‐specific public health strategies. Those who belong to Generation X (currently aged between 44 and 59), may benefit from individualised intervention approaches, particularly given they are likely to have increasingly complex medical and psychosocial needs related to prolonged meth/amphetamine use [e.g. mental health, cardiovascular disease (CVD), criminal justice involvement and homelessness] [61]. Some treatment outcomes are promising among older adults, with people aged 50+ having the highest percentage of completed meth/amphetamine‐related treatment episodes of any age group in Australia [62]. However, there is limited access to specialised services catering for older adults. People who use meth/amphetamine may avoid accessing services because of stigma and doubts about treatment effectiveness [63]. Reducing drug‐related stigma among health professionals is key to enhancing engagement in meth/amphetamine treatment. This includes correcting misinformation about people who use meth/amphetamines (e.g. that they are dangerous and unpredictable), promoting compassionate and non‐judgmental care and providing evidence‐based education on the individual needs of those who use meth/amphetamine [64, 65]. Community education campaigns, such as Australia's Cracks in the Ice program, are important for providing evidence based information to reduce stigmatising views [59]. There remains a lack of research regarding effective engagement strategies and methods to reduce barriers to meth/amphetamine treatment among older people.

Previous Australian research has suggested that meth/amphetamine‐related CVD deaths are prominent among people who use meth/amphetamine [7]. As Generation X ages, the risk of CVD is likely to increase because of the cumulative impact of regular meth/amphetamine use, in combination with the natural increase in the risk of CVD as a person ages [37, 66]. This suggests that increased access to screening and early management of CVD among older people who use meth/amphetamine may be useful to reduce CVD deaths. Community‐based CVD outreach screening programs have demonstrated effectiveness in removing disjointed transitions between assessment and follow‐up, as well as improving CVD risk factors. These programs have successfully reached marginalised populations, including those from culturally and linguistically diverse backgrounds, from rural areas and people who experience homelessness [67, 68, 69]. Outreach screening may potentially improve early detection and intervention for people who use meth/amphetamine.

### Limitations

One of the major limitations of this study is that the period was confined to 20 years. Accordingly, the APC‐I analysis could not include each generation's whole lifespan, limiting the explanatory power between generations. Mortality research using the NCIS faces limitations in case classification because it is restricted by the information provided in the cause of death and investigative reports. The complexity and length of coronial investigations mean that recorded deaths in the later years of this study may be underestimated, potentially leading to lower effect sizes in the last period. Although this study included all meth/amphetamine‐related deaths reported to Australian coroners during the study period, systematic biases such as under‐reporting, misclassification and changing practices in toxicology screening may have influenced our results. For instance, evolving forensic practices over the 20‐year study period might have increased the likelihood of identifying meth/amphetamine involvement in more recent deaths, potentially inflating observed period effects. The APC‐I model in our analysis may overfit the data, but is valuable for exploring complex interactions and cohort effects in population‐level mortality trends without generalising beyond the data. The APC‐I model's use of Poisson regression and overdispersion within the available aggregate mortality data might theoretically lead to less reliable standard error estimation and inflated significance values. However, as noted above, this is less critical for this study because we relied on population rather than sample data, making issues of statistical inference, such as inflated significance values, less relevant [70, 71]. The analysis did not control for a recorded history of injecting drug use, which is a significant risk factor for mortality among people who use drugs [72] and is estimated to be prevalent among 44% of this sample [7].

## CONCLUSION

Meth/amphetamine‐related mortality in Australia has undergone significant shifts between 2001 and 2020. The APC‐I analysis shows varying mortality trends across generations, with Baby Boomers facing rising mortality risk with age, Generation X maintaining a high but stable risk, and Millennials and Generation Z seeing a decreasing risk. The higher mortality risk seen among Generation X may in part be attributed to the lasting impact of drug market changes experienced by this cohort during their formative years. The decreased risk among Millennials and Generation Z may point to a broader trend of decreasing risk behaviours among younger people and the impact of stigma reducing meth/amphetamine initiation. Understanding these cohort clusters can help enhance the effectiveness of strategies aimed at reducing meth/amphetamine‐related harm, and guide treatment and harm reduction resources.

## AUTHOR CONTRIBUTIONS

**Oisin Stronach:** Data curation (lead); formal analysis (lead); methodology (supporting); visualization (lead); writing—original draft (lead); writing—review and editing (lead). **Paul Dietze:** Conceptualization (supporting); methodology (supporting); supervision (supporting); writing—review and editing (supporting). **Michael Livingston:** Conceptualization (supporting); methodology (supporting); supervision (supporting); writing—review and editing (supporting). **Amanda Roxburgh:** Conceptualization (lead); funding acquisition (lead); methodology (lead); project administration (lead); supervision (lead); writing—review and editing (supporting).

## DECLARATION OF INTERESTS

O.S. is supported by an Australian Government Research Training Program Scholarship; A.R. is supported by the National Health and Medical Research Council (APP1173505).

## Supporting information


**Table S1.** Definitions for methamphetamine‐related death categories.
**Figure S1.** Comparing patterns of age effects on meth/amphetamine‐related mortality rates based on a partial AP Poisson model (a) and a partial AP Negative Binomial model (b).
**Figure S2.** Meth/amphetamine‐related crude mortality rates per 100 000 population by age, period and cohort for a) unintentional drug toxicity, b) intentional self‐harm, c) unintentional injury and d) natural causes.
**Table S2.** APC‐I global likelihood ratio test results.
**Table S3.1.** Coefficient estimates of age‐by‐period interaction terms in the APC‐I model of meth/amphetamine‐related unintentional drug toxicity mortality.
**Table S3.2.** Coefficient estimates of age‐by‐period interaction terms in the APC‐I model of meth/amphetamine‐related intentional self‐harm mortality.
**Table S3.3.** Coefficient estimates of age‐by‐period interaction terms in the APC‐I model of meth/amphetamine‐related unintentional injury mortality.
**Table S3.4.** Coefficient estimates of age‐by‐period interaction terms in the APC‐I model of meth/amphetamine‐related natural causes mortality.
**Figure S3**. Estimated intra‐cohort deviations of meth/amphetamine‐related unintentional drug toxicity, intentional self‐harm, unintentional injury, and natural causes.

## Data Availability

Due to the sensitivities of coronial data, our ethics approval and data access agreement we are prohibited from sharing this data outside the research group.
