# Supplementary material for: An age–period–cohort–interaction analysis of meth/amphetamine‐related deaths in Australia, 2001–2020
Source: Addiction. 2025 Jul 1;120(10):2032–43. doi: 10.1111/add.70100 (PMC12426365; doi:10.1111/add.70100)
Supplement: Supplementary file 1 — Table S1. Definitions for methamphetamine‐related death categories. Figure S1. Comparing patterns of age effects on meth/amphetamine‐related mortality rates based on a partial AP Poisson model (a) and a partial AP Negative Binomial model (b). Figure S2. Meth/amphetamine‐related crude mortality rates per 100 000 population by age, period and cohort for a) unintentional drug toxicity, b) intentional self‐harm, c) unintentional injury and d) natural causes. Table S2. APC‐I global likelihood ratio test results. Table S3.1. Coefficient estimates of age‐by‐period interaction terms in the APC‐I model of meth/amphetamine‐related unintentional drug toxicity mortality. Table S3.2. Coefficient estimates of age‐by‐period interaction terms in the APC‐I model of meth/amphetamine‐related intentional self‐harm mortality. Table S3.3. Coefficient estimates of age‐by‐period interaction terms in the APC‐I model of meth/amphetamine‐related unintentional injury mortality. Table S3.4. Coefficient estimates of age‐by‐period interaction terms in the APC‐I model of meth/amphetamine‐related natural causes mortality. Figure S3. Estimated intra‐cohort deviations of meth/amphetamine‐related unintentional drug toxicity, intentional self‐harm, unintentional injury, and natural causes. [file ADD-120-2032-s001.docx]

**Supplementary Materials**

**Table of Contents**

[Supplementary Table 1. Definitions for methamphetamine‐related death categories. 1](#_Toc184894569)

[Justification for Using an APC-I Poisson Model 2](#_Toc184894570)

[Supplementary Figure 1. Comparing patterns of age effects on meth/amphetamine-related mortality rates based on a partial AP Poisson model (a) and a partial AP Negative Binomial model (b). 5](#_Toc184894571)

[Supplementary Figure 2. Meth/amphetamine-related crude mortality rates per 100,000 population by age, period and cohort for a) unintentional drug toxicity, b) intentional self-harm, c) unintentional injury and d) natural causes. 6](#_Toc184894572)

[Supplementary Table 2. APC-I global likelihood ratio test results. 7](#_Toc184894573)

[Supplementary Table 3.1. Coefficient estimates of age-by-period interaction terms in the APC-I model of meth/amphetamine-related unintentional drug toxicity mortality. 7](#_Toc184894574)

[Supplementary Table 3.2. Coefficient estimates of age-by-period interaction terms in the APC-I model of meth/amphetamine-related intentional self-harm mortality. 8](#_Toc184894575)

[Supplementary Table 3.3. Coefficient estimates of age-by-period interaction terms in the APC-I model of meth/amphetamine-related unintentional injury mortality. 8](#_Toc184894576)

[Supplementary Table 3.4. Coefficient estimates of age-by-period interaction terms in the APC-I model of meth/amphetamine-related natural causes mortality. 9](#_Toc184894577)

[Age-by-period interaction interpretation 9](#_Toc184894578)

[Supplementary Figure 3. Estimated intra-cohort deviations of meth/amphetamine-related unintentional drug toxicity, intentional self-harm, unintentional injury, and natural causes. 11](#_Toc184894579)

[Intra-cohort deviation interpretation 12](#_Toc184894580)

[References 13](#_Toc184894581)

#

# Supplementary Table 1. Definitions for methamphetamine‐related death categories.

| Cause of death | Definition |
| --- | --- |
| Unintentional drug toxicity | Unintentional drug toxicity was defined as cases where the coroner listed methamphetamine or amphetamine toxicity as the underlying cause of death or cases with generic phrases like “drug overdose”, “drug toxicity”, “mixed drug toxicity”, etc., listed in the underlying cause of death, and methamphetamine was present in toxicology at death. |
| Intentional self-harm | Intentional self-harm deaths are those where the coroner determined the deceased intentionally self-inflicted harm with the intention of deliberately ending their life, or where the deceased intentionally self-inflicted harm, regardless of whether the anticipated outcome was death, and methamphetamine was present in toxicology at death. |
| Unintentional injury | Unintentional injury deaths are those where the coroner has determined the underlying cause of death was unintentional and involved an event causing injury and methamphetamine was present in toxicology at death. |
| Natural causes | Natural cause deaths are those where the coroner has determined the underlying cause of death was attributable to a pre-existing disease, and contributory causes included methamphetamine. Natural cause deaths were further classified from the medical cause of death into categories (e.g. circulatory system diseases) based on the International Classification of Diseases (ICD, 10th revision) (World Health Organization, 2019). |
| Assault | Assault cases were those where the coroner identified assault was the cause of death and methamphetamine was present in toxicology at death. |

# Justification for Using an APC-I Poisson Model

This study employed a Poisson model due to the limited degrees of freedom inherent in the aggregate-level age-mortality data, which prevented the application of an APC-I approach using a negative binomial model. In the methamphetamine mortality data, each age-period-cohort configuration corresponds to a single observation (i.e., a single age-period-cohort-specific mortality rate). This means that the number of parameters estimated equals the number of observations (60 parameters for 60 observations), resulting in zero residual degrees of freedom. As a result, estimating age main effects, period main effects, and age-by-period interactions simultaneously in an APC-I framework becomes challenging. The saturated model fits the data perfectly, leaving no residual variation to assess. Therefore, there are no residuals to exhibit overdispersion. Although a negative binomial model could theoretically accommodate the overdispersion present in the mortality data, the lack of degrees of freedom makes it infeasible to fit an APC-I negative binomial model. By contrast, the Poisson model, which requires only one parameter rather than two, provides an additional degree of freedom, thus enabling the specification of an APC-I model with the available aggregate mortality data.

Although mortality data frequently exhibits overdispersion, causing a Poisson model to underestimate standard errors and inflate the perceived significance of regression parameters, this issue is less important for this study. (1) Unlike sample-based research, the current study uses population data, where the primary goal is to estimate the age, period and cohort effects on methamphetamine-related mortality in Australia. Under these circumstances, standard errors and statistical significance are included only as a reference. (2) Since the results reflect population parameters rather than sample statistics, the focus should remain on the effect sizes rather than the standard errors and significance. (3, 4)

For the Age-Period (AP) model (a partial model incorporating the age and period main effects without controlling for age-by-period interactions), there are positive residual degrees of freedom (44 degrees of freedom, calculated as 60 observations minus 16 parameters). Overdispersion was tested in these models by calculating the overdispersion ratio, which was found to be significantly greater than 1 for all causes of death. To further elaborate on this issue and address the concern of overdispersion, AP models fitted using Poisson and negative binomial distributions were compared. Supplementary Figure 1 shows that while the negative binomial model produces larger standard errors than the Poisson model due to overdispersion, the overall age-mortality patterns, remain broadly similar across the two modelling approaches after accounting for period effects.

The APC-I model’s reliance on a Poisson model might theoretically lead to less reliable standard error estimation. However, this is less critical for this study as we relied on population rather than sample data, making issues of statistical inference, such as inflated significance values, less relevant. Our primary interest lies in understanding the magnitude and direction of age, period, and cohort effects, which remain robust to these methodological constraints. In the AP models (without interaction), residual degrees of freedom allowed us to test for overdispersion, the introduction of a negative binomial distribution confirmed that larger standard errors result from accounting for overdispersion. Nonetheless, the main age and period patterns remain broadly consistent, suggesting that the presence of overdispersion does not alter the observed underlying mortality trends.

# Supplementary Figure 1. Comparing patterns of age effects on meth/amphetamine-related mortality rates based on a partial AP Poisson model (a) and a partial AP Negative Binomial model (b).

**
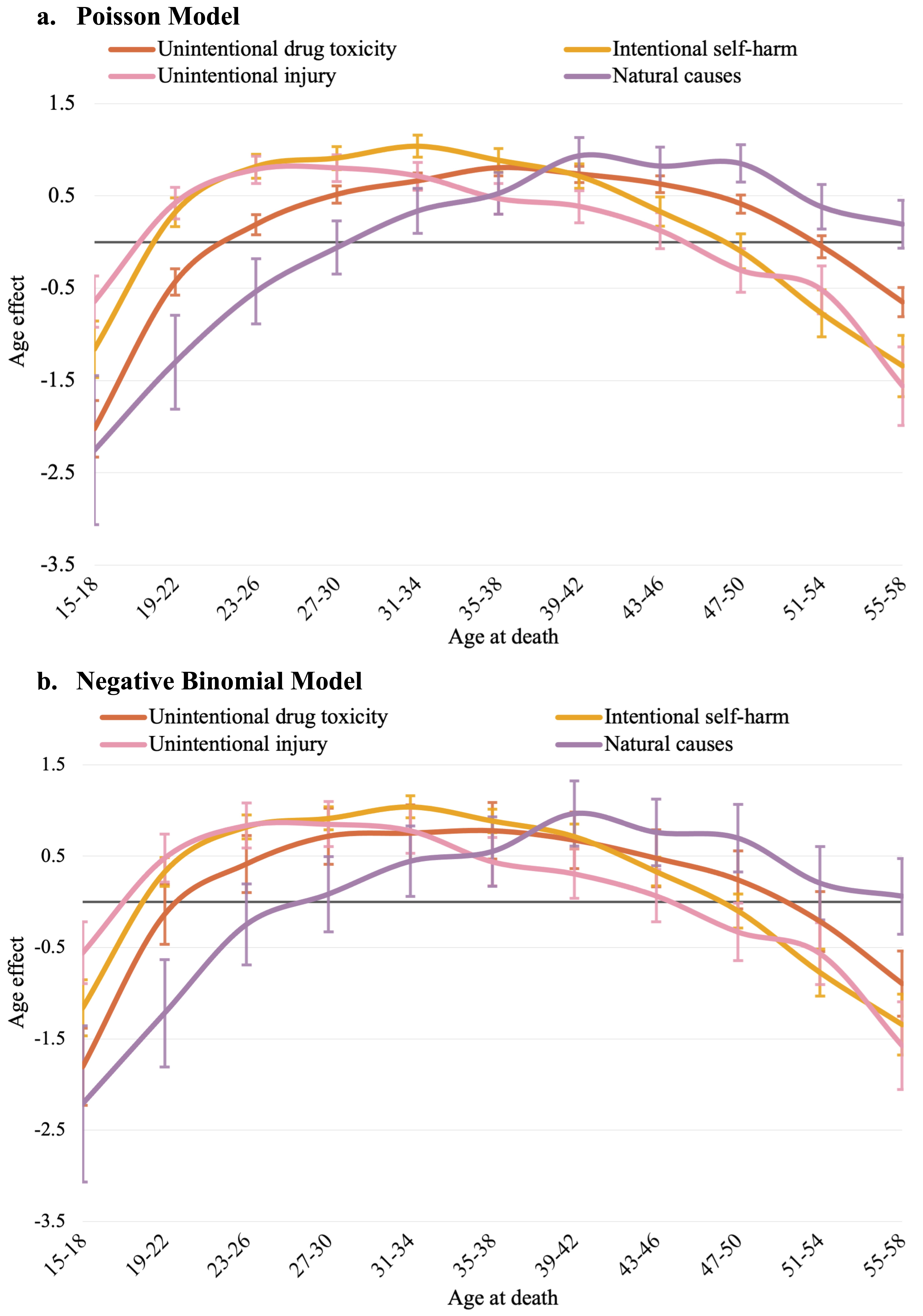
**

# Supplementary Figure 2. Meth/amphetamine-related crude mortality rates per 100,000 population by age, period and cohort for a) unintentional drug toxicity, b) intentional self-harm, c) unintentional injury and d) natural causes.


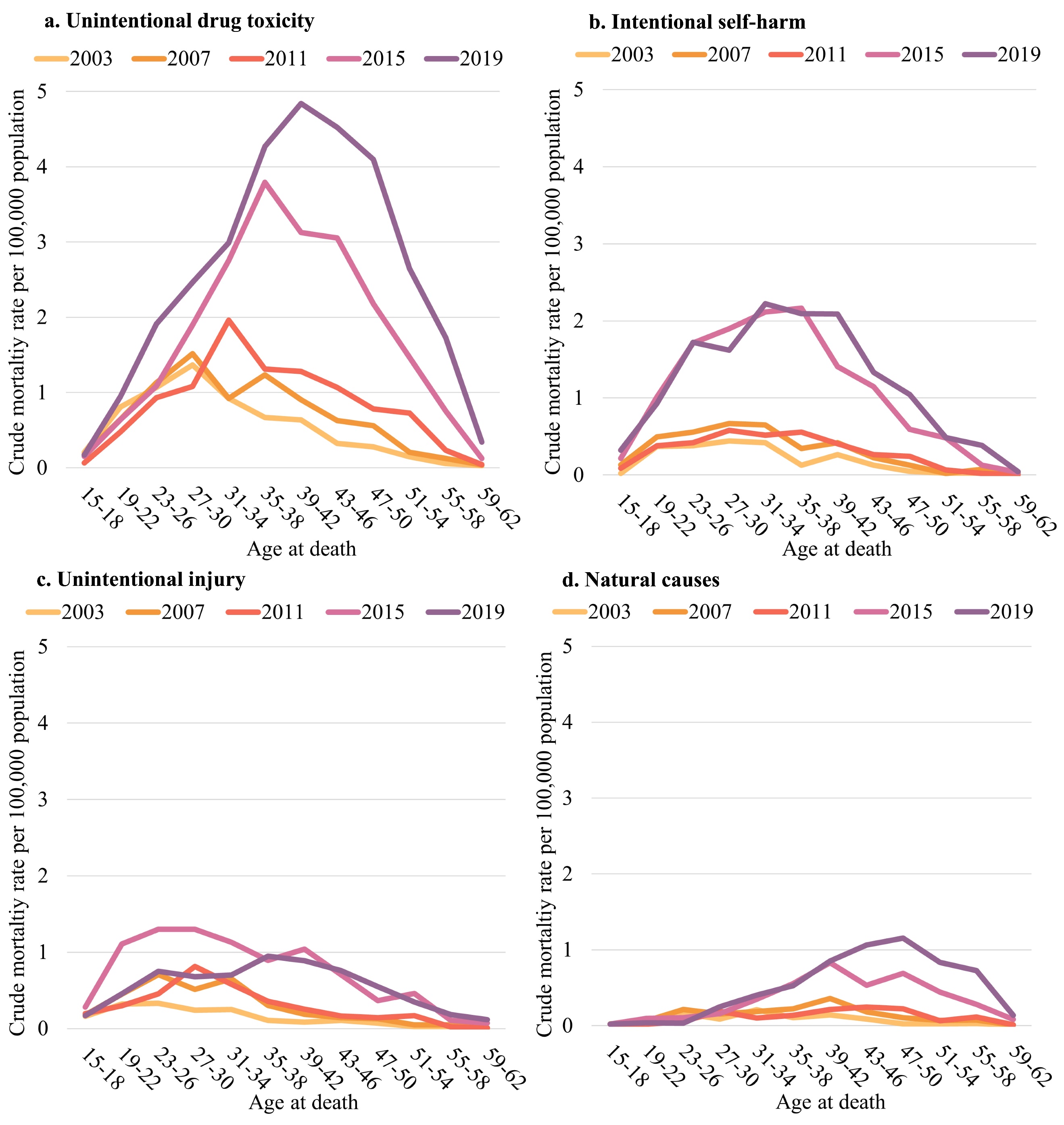


# Supplementary Table 2. APC-I global likelihood ratio test results.

| Cause of death | Deviance | df | Sig |
| --- | --- | --- | --- |
| Unintentional drug toxicity | 343·92 | 44 | <0·001 |
| Intentional self-harm | 71·11 | 44 | 0·006 |
| Natural causes | 113·91 | 44 | <0·001 |
| Unintentional injury | 120·94 | 44 | <0·001 |

Note: Table figures represent likelihood ratio tests wherein a model with age and period terms was tested against a model that included age, period, and age-by-period terms·

# Supplementary Table 3.1. Coefficient estimates of age-by-period interaction terms in the APC-I model of meth/amphetamine-related unintentional drug toxicity mortality.

|  | Period | | | | | | | | | |  |
| --- | --- | --- | --- | --- | --- | --- | --- | --- | --- | --- | --- |
|  | 2001 - 2004 | | 2005 - 2008 | | 2009 - 2012 | | 2013 - 2016 | | 2017 - 2020 | |  |
| Age group | Coef· | Sig· | Coef· | Sig· | Coef· | Sig· | Coef· | Sig· | Coef· | Sig· | **Generation** |
| 15 - 18 | 1·11 | *** | 0·66 | * | -0·54 |  | -0·45 |  | -0·78 | * | *Generation Z* |
| 19 - 22 | 0·91 | *** | 0·39 | * | -0·10 |  | -0·57 | *** | -0·63 | *** |  |
| 23 - 26 | 0·64 | *** | 0·40 | ** | 0·02 |  | -0·59 | *** | -0·48 | *** | *Millennials* |
| 27 - 30 | 0·59 | *** | 0·39 | *** | -0·13 |  | -0·33 | *** | -0·52 | *** |  |
| 31 - 34 | 0·14 |  | -0·16 |  | 0·41 | *** | -0·01 |  | -0·38 | *** |  |
| 35 - 38 | -0·23 |  | 0·08 |  | -0·04 |  | 0·26 | ** | -0·07 |  |  |
| 39 - 42 | -0·19 |  | -0·14 |  | 0·03 |  | 0·16 |  | 0·14 |  | *Generation X* |
| 43 - 46 | -0·60 | ** | -0·24 |  | 0·11 |  | 0·40 | *** | 0·34 | *** |  |
| 47 - 50 | -0·55 | * | -0·15 |  | 0·00 |  | 0·26 | * | 0·44 | *** |  |
| 51 - 54 | -0·69 | * | -0·63 | * | 0·43 | * | 0·37 | ** | 0·51 | *** |  |
| 55 - 58 | -0·92 |  | -0·41 |  | 0·04 |  | 0·45 | * | 0·83 | *** | *Baby Boomers* |
| 59 - 62 | -0·20 |  | -0·20 |  | -0·23 |  | 0·04 |  | -0·59 | *** |  |

*p<·05; **p<·01; ***p<·001. Given that our analysis utilises population data for all Australian methamphetamine-related deaths and statistical inferences are not being made outside of the study period, statistical significance is included solely for reference purposes. We focus on the effect sizes, interpreting them as population parameters rather than sample estimates

# Supplementary Table 3.2. Coefficient estimates of age-by-period interaction terms in the APC-I model of meth/amphetamine-related intentional self-harm mortality.

|  | Period | | | | | | | | | |  |
| --- | --- | --- | --- | --- | --- | --- | --- | --- | --- | --- | --- |
|  | 2001 - 2004 | | 2005 - 2008 | | 2009 - 2012 | | 2013 - 2016 | | 2017 - 2020 | |  |
| Age group | Coef· | Sig· | Coef· | Sig· | Coef· | Sig· | Coef· | Sig· | Coef· | Sig· | **Generation** |
| 15 - 18 | -0·52 |  | 0·55 |  | 0·24 |  | -0·22 |  | -0·04 |  | *Generation Z* |
| 19 - 22 | 0·62 | * | 0·22 |  | 0·08 |  | -0·31 | * | -0·61 | *** |  |
| 23 - 26 | 0·37 |  | 0·06 |  | -0·10 |  | -0·06 |  | -0·27 | * | *Millennials* |
| 27 - 30 | 0·37 |  | 0·11 |  | 0·09 |  | -0·10 |  | -0·47 | *** |  |
| 31 - 34 | 0·28 |  | 0·03 |  | -0·08 |  | -0·03 |  | -0·20 |  |  |
| 35 - 38 | -0·54 |  | -0·25 |  | 0·36 |  | 0·35 | * | 0·09 |  |  |
| 39 - 42 | 0·15 |  | -0·09 |  | 0·01 |  | -0·12 |  | 0·05 |  | *Generation X* |
| 43 - 46 | -0·09 |  | -0·23 |  | 0·07 |  | 0·16 |  | 0·09 |  |  |
| 47 - 50 | -0·60 |  | -0·26 |  | 0·49 |  | 0·01 |  | 0·36 |  |  |
| 51 - 54 | -0·32 |  | -1·05 |  | 0·09 |  | 0·74 | * | 0·53 |  |  |
| 55 - 58 | 0·06 |  | 0·37 |  | -0·66 |  | -0·32 |  | 0·55 |  | *Baby Boomers* |
| 59 - 62 | 0·23 |  | 0·53 |  | -0·57 |  | -0·11 |  | 0·08 |  |  |

*p<·05; **p<·01; ***p<·001. Given that our analysis utilises population data for all Australian methamphetamine-related deaths and statistical inferences are not being made outside of the study period, statistical significance is included solely for reference purposes. We focus on the effect sizes, interpreting them as population parameters rather than sample estimates

# Supplementary Table 3.3. Coefficient estimates of age-by-period interaction terms in the APC-I model of meth/amphetamine-related unintentional injury mortality.

|  | Period | | | | | | | | | |  |
| --- | --- | --- | --- | --- | --- | --- | --- | --- | --- | --- | --- |
|  | 2001 - 2004 | | 2005 - 2008 | | 2009 - 2012 | | 2013 - 2016 | | 2017 - 2020 | |  |
| Age group | Coef· | Sig· | Coef· | Sig· | Coef· | Sig· | Coef· | Sig· | Coef· | Sig· | **Generation** |
| 15 - 18 | 0·74 | * | 0·27 |  | 0·25 |  | -0·47 |  | -0·80 | ** | *Generation Z* |
| 19 - 22 | 0·55 | * | 0·33 |  | -0·20 |  | 0·03 |  | -0·70 | *** |  |
| 23 - 26 | 0·27 |  | 0·46 | * | -0·09 |  | -0·12 |  | -0·51 | *** | *Millennials* |
| 27 - 30 | -0·01 |  | 0·17 |  | 0·52 | ** | -0·09 |  | -0·58 | *** |  |
| 31 - 34 | 0·06 |  | 0·44 | * | 0·21 |  | -0·20 |  | -0·51 | *** |  |
| 35 - 38 | -0·39 |  | 0·09 |  | 0·14 |  | -0·03 |  | 0·19 |  |  |
| 39 - 42 | -0·46 |  | -0·19 |  | -0·01 |  | 0·32 |  | 0·33 |  | *Generation X* |
| 43 - 46 | 0·02 |  | -0·27 |  | -0·24 |  | 0·13 |  | 0·37 | * |  |
| 47 - 50 | -0·09 |  | -0·04 |  | -0·06 |  | -0·19 |  | 0·38 |  |  |
| 51 - 54 | -0·72 |  | -0·64 |  | 0·55 |  | 0·47 |  | 0·34 |  |  |
| 55 - 58 | 0·19 |  | 0·21 |  | -0·66 |  | -0·21 |  | 0·47 |  | *Baby Boomers* |
| 59 - 62 | -0·16 |  | -0·84 |  | -0·39 |  | 0·36 |  | -1·03 | ** |  |

*p<·05; **p<·01; ***p<·001. Given that our analysis utilises population data for all Australian methamphetamine-related deaths and statistical inferences are not being made outside of the study period, statistical significance is included solely for reference purposes. We focus on the effect sizes, interpreting them as population parameters rather than sample estimates

# Supplementary Table 3.4. Coefficient estimates of age-by-period interaction terms in the APC-I model of meth/amphetamine-related natural causes mortality.

|  | Period | | | | | | | | | |  |
| --- | --- | --- | --- | --- | --- | --- | --- | --- | --- | --- | --- |
|  | 2001 - 2004 | | 2005 - 2008 | | 2009 - 2012 | | 2013 - 2016 | | 2017 - 2020 | |  |
| Age group | Coef· | Sig· | Coef· | Sig· | Coef· | Sig· | Coef· | Sig· | Coef· | Sig· | **Generation** |
| 15 - 18 | 0·86 |  | 0·25 |  | 0·39 |  | -0·68 |  | -0·83 |  | *Generation Z* |
| 19 - 22 | 0·81 |  | 0·57 |  | -0·45 |  | 0·08 |  | -1·01 |  |  |
| 23 - 26 | 1·38 | *** | 1·06 | ** | -0·10 |  | -0·53 |  | -1·82 | ** | *Millennials* |
| 27 - 30 | 0·22 |  | 0·22 |  | 0·59 |  | -0·67 | * | -0·36 |  |  |
| 31 - 34 | 0·75 | * | 0·07 |  | -0·37 |  | -0·22 |  | -0·22 |  |  |
| 35 - 38 | -0·03 |  | 0·13 |  | -0·18 |  | 0·14 |  | -0·06 |  |  |
| 39 - 42 | -0·15 |  | 0·18 |  | -0·17 |  | 0·13 |  | 0·01 |  | *Generation X* |
| 43 - 46 | -0·41 |  | -0·24 |  | 0·22 |  | -0·06 |  | 0·48 | * |  |
| 47 - 50 | -1·41 |  | -0·44 |  | 0·44 |  | 0·52 |  | 0·89 | *** |  |
| 51 - 54 | -0·88 |  | -0·40 |  | -0·32 |  | 0·55 |  | 1·04 | *** |  |
| 55 - 58 | -0·79 |  | -0·37 |  | 0·24 |  | 0·06 |  | 0·86 | ** | *Baby Boomers* |
| 59 - 62 | -0·34 |  | -1·03 |  | -0·30 |  | 0·67 |  | -1·01 | ** |  |

*p<·05; **p<·01; ***p<·001. Given that our analysis utilises population data for all Australian methamphetamine-related deaths and statistical inferences are not being made outside of the study period, statistical significance is included solely for reference purposes. We focus on the effect sizes, interpreting them as population parameters rather than sample estimates

# Age-by-period interaction interpretation

Each age-by-period interaction term is interpreted as the variation from the main age and period effects for a specific cohort at a given age or within a particular period. For example, in Supplementary Table 3.1, for unintentional drug toxicity deaths, the estimated age-by-period interaction term for 15-18 year olds in 2001 – 2004 (1986 cohort) was 1·11, which suggests that the 1986 cohort between ages 15-18 experience a 200% (exp(1·11)-1) increase in rates of meth/amphetamine-related unintentional drug toxicity compared to the predicted mortality rate determined by age and period effects. Supplementary Tables 3.1 to 3.4 present the estimates of age-by-period interaction terms for each cause of death, where the diagonal entries in each table reflect the deviations of each cohort over the study period, with age and period main effects held constant. For unintentional drug toxicity, some significant interactions were observed that generally remained consistent along the diagonals. For example, Generation X generally had higher than average odds of unintentional drug toxicity related mortality than other cohorts and remained higher than average into the late 2010s as these cohorts aged. Between 2017 – 2020, significant negative age-by-period interaction terms were observed for people aged between 19–30 for intentional self-harm deaths and people aged between 15–34 for unintentional injury deaths, suggesting lower-than-average odds of mortality for these cohorts in this period. Between 2017 – 2020, significant positive age-by-period interaction terms were observed for people aged between 43–58 for natural cause deaths, suggesting higher than average odds of mortality for these cohorts in this period.

Supplementary Figure 3. Estimated intra-cohort deviations of meth/amphetamine-related unintentional drug toxicity, intentional self-harm, unintentional injury, and natural causes.**
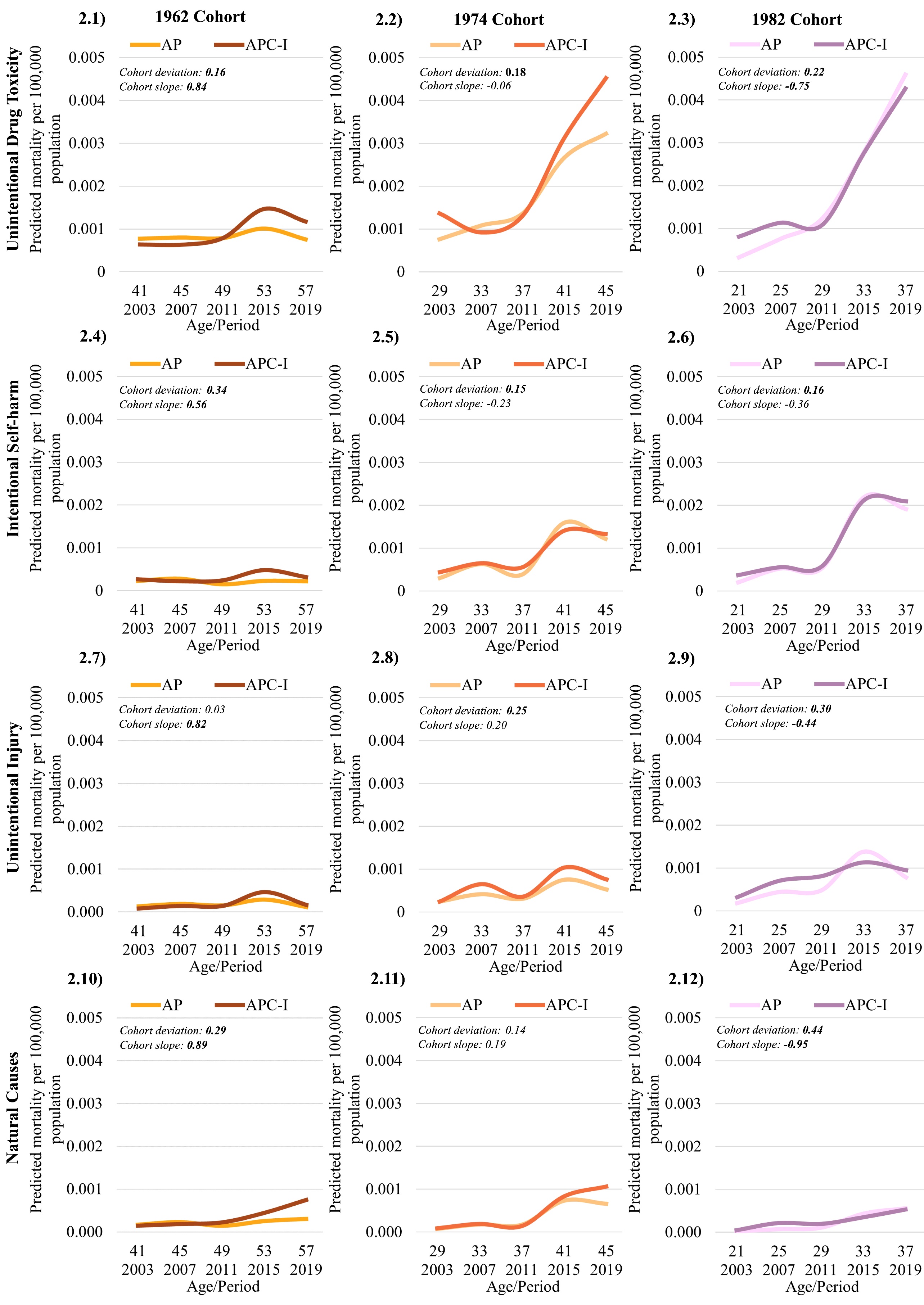
**

# Intra-cohort deviation interpretation

To better illustrate these life-course dynamics, we graphically present a late Baby Boomer cohort (1962), a middle Generation X cohort (1974) and an early Millennial cohort (1982), which shows these cohorts’ life-course patterns for the different causes of death. Figure 4 shows the expected mortality rates for different cohorts over their lifetimes, as determined by the estimated age-by-period interaction terms (labelled as "APC-I"), as well as the mortality rates determined by just the age and period terms (labelled as "AP"). The difference between the APC-I and AP lines indicates the cohort effect at a given age and at a certain time point in the cohort’s life-course trajectory. The analysis reveals that the 1962 cohort unintentional drug toxicity, intentional self-harm, and natural causes deaths have significant cohort effects and significant positive intra-cohort slopes. This suggests that this cohort has a higher mortality risk and that cohort effects generally increase between ages 41 and 57, particularly in 2015. In the 1974 cohort, while there are significant cohort effects for unintentional drug toxicity, intentional self-harm, and unintentional injury deaths, the intra-cohort slopes do not show significant changes. This indicates that this cohort experiences a higher mortality risk, and the cohort effect remains stable between ages 29 and 45. In contrast, the 1982 cohort has significant cohort effects for all causes of death and significant negative intra-cohort slopes for unintentional drug toxicity, unintentional injury, and natural causes of death. This implies that this cohort has a higher mortality risk, and the cohort effects decrease between ages 29 and 45. These life-course dynamics results should be taken with caution as the study period is only 20 years and does not capture people’s whole lives. This may explain why we see positive intra-cohort deviations within early cohorts and negative intra-cohort deviations within later cohorts.

# References

1. Cairns AJG, Blake D, Dowd K, Coughlan GD, Epstein D, Ong A, Balevich I. A Quantitative Comparison of Stochastic Mortality Models Using Data From England and Wales and the United States. North American Actuarial Journal. 2009;13(1):1-35.

2. Wasserstein RL, Lazar NA. The ASA Statement on p-Values: Context, Process, and Purpose. The American Statistician. 2016;70(2):129-33.

3. Alexander N. What’s more general than a whole population? Emerging Themes in Epidemiology. 2015;12(1):11.

4. Berk RA, Western B, Weiss RE. Statistical Inference for Apparent Populations. Sociological Methodology. 1995;25:421-58.
